# Building evidence into youth health policy: a case study of the *Access 3* knowledge translation forum

**DOI:** 10.1186/s12961-022-00845-y

**Published:** 2022-04-20

**Authors:** Daniel Waller, Fiona Robards, Carmen Huckel Schneider, Lena Sanci, Katharine Steinbeck, Sally Gibson, Tim Usherwood, Catherine Hawke, Stephen Jan, Marlene Kong, Melissa Kang

**Affiliations:** 1 Faculty of Health, University of Technology Sydney, Ultimo, Australia; 2 Faculty of Medicine and Health, University of Sydney, Camperdown, Australia; 3 Faculty of Medicine, Dentistry and Health Sciences, University of Melbourne, Parkville, Australia; 4 New South Wales Ministry of Health, St Leonards, Australia; 5 The George Institute for Global Health, University of New South Wales, Sydney, Australia; 6 The Kirby Institute, University of New South Wales, Sydney, Australia; 7 School of Public Health, Faculty of Health, University of Technology Sydney, Level 8, Building 10, 235 Jones Street, Ultimo, NSW 2007 Australia

**Keywords:** Youth, Health, Policy, Knowledge translation, Implementation science, Policy translation

## Abstract

**Background:**

Effective integration of evidence and youth perspectives into policy is crucial for supporting the future health and well-being of young people. The aim of this project was to translate evidence from the *Access 3* project to support development of a new state policy on youth health and well-being within New South Wales (NSW), Australia. Ensuring the active contribution of young people within policy development was a key objective of the knowledge translation (KT) process.

**Methods:**

The KT activity consisted of a 1-day facilitated forum with 64 purposively sampled stakeholders. Participants included eight young people, 14 policy-makers, 15 academics, 22 clinicians or managers from NSW health services, four general practitioners and one mental health service worker. Research to be translated came from the synthesized findings of the NSW *Access 3* project. The design of the forum included stakeholder presentations and group workshops, guided by the 2003 Lavis et al. KT framework that was improved by the Grimshaw et al. KT framework in 2012. Members of the *Access 3* research team took on the role of knowledge brokers throughout the KT process. Participant satisfaction with the workshop was evaluated using a brief self-report survey. Policy uptake was determined through examination of the subsequent NSW Youth Health Framework 2017–2024.

**Results:**

A total of 25 policy recommendations were established through the workshop, and these were grouped into six themes that broadly aligned with the synthesized findings from the *Access 3* project. The six policy themes were (1) technology solutions, (2) integrated care and investment to build capacity, (3) adolescent health checks, (4) workforce, (5) youth participation and (6) youth health indicators. Forum members were asked to vote on the importance of individual recommendations. These policy recommendations were subsequently presented to the NSW Ministry of Health, with some evidence of policy uptake identified. The majority of participants rated the forum positively.

**Conclusions:**

The utilization of KT theories and active youth engagement led to the successful translation of research evidence and youth perspectives into NSW youth health policy. Future research should examine the implementation of policy arising from these KT efforts.

**Supplementary Information:**

The online version contains supplementary material available at 10.1186/s12961-022-00845-y.

## Contributions to literature

This paper:

- presents an inclusive approach to KT through a project focused on development of youth health policy;
- demonstrates how government–academic partnerships can successfully build evidence-informed policy;
- shows that using a theoretical approach to KT assists in the development of evidence-based policy recommendations;
- shows that including young people and other relevant stakeholders assists in the development of evidence-based policy recommendations;
- will be of interest to domestic and international policy-makers developing and implementing youth health policies.

## Background

The period of adolescence and young adulthood is a time where lifelong health behaviours and service engagement patterns are formed and several unique health risks emerge [1, 2]. Improving health during adolescence is understood to provide a “triple dividend” in that this allows for optimal youth development, improves long-term trajectories of health, and provides the healthiest start possible for the subsequent generation [1]. The time of adolescence and young adulthood thus presents a critical opportunity for policy-makers to protect the current and future health of societies through the creation and adoption of evidence-informed policies focused specifically on youth health.

Young people face several health barriers including, but not limited to, difficulties accessing health services, lack of knowledge and experience in navigating healthcare systems, impacts of restrictive legislation and concerns over confidentiality, stigma and cost [1, 3]. Training in adolescent health is variable, as are the knowledge, attitudes and skills of clinicians working with young people [3]. Consequently, young people’s health needs may not be fully met [1]. Health policy can help address these issues by building a framework for the health system that promotes young people’s access, provides quality health worker training and delivers adolescent-responsive services [1].

Health policy shapes health systems and services, directs funding for infrastructure and resources, helps to determine key health priorities, and supports innovation and implementation efforts [4, 5]. Evidence-informed health policy seeks to increase the use of evidence (derived from data and research insights, expert consensus and patient lived experiences) to guide decision-making on the merits, design and implementation of policy actions [5]. The use of scientifically robust evidence (e.g. evidence derived from systematic reviews, randomized controlled trials and large-scale cohort studies) aids policy-makers in identifying policy priorities, weighing the costs and benefits of public investments, determining the most effective policy steps to enact (and how), and identifying whether more efficient alternatives exist [5]. Together, this highlights the importance of building high-quality translatable evidence to support the development and implementation of effective youth health policy.

However, whilst the importance of evidence to inform health policy is well acknowledged, the failure to translate evidence into policy continues to be an area of concern [5–10]. Evidence derived from research studies often fails to determine or reach policy audiences despite decades of calls for stronger links between research and policy action [8–10]. Furthermore, the engagement of young people as a method to generate evidence to identify important health issues and policy needs has often been underutilized [2, 11–14].

Knowledge translation (KT) refers to formal attempts to ensure that stakeholders are aware of and utilize relevant evidence to guide decisions [6, 7]. In the current setting, focused KT efforts are required to translate evidence gained from research studies and young people’s lived experiences into policy actions that promote the health and well-being of young people. Utilizing established KT frameworks such as those provided by Lavis et al. [6] and Grimshaw et al. [7] assists researchers and policy-makers in building effective collaborations and provides a common language for the development of evidence-informed health policies [6, 7, 15].

## Policy-setting

Australia’s health system includes a diverse range of healthcare providers and complex funding arrangements [17, 18]. The federal government funds Australia’s universal health insurance (Medicare), the Pharmaceutical Benefits Scheme (PBS) and Primary Health Networks (PHNs). PHNs are geographically based administrative organizations that support providers of primary care and liaise with hospitals and other providers to improve health system efficiency across their regions [17, 18]. Australian states are responsible for policy development in public and population health, and public hospital and community health funding and management [17, 18]. The Australian primary care sector is dominated by general practice, which operates as private businesses, albeit with the bulk of their income sourced on a fee-for-service basis, reimbursed by Medicare [10, 11]. Importantly, government policy cannot direct clinical services delivered by general practice but can influence it through federally controlled Medicare rebate incentives and other occasional targeted payments. The federal government also funds Headspace, a national youth mental health foundation that provides services for young people aged 12 to 25 years.

New South Wales (NSW) is the most populous state in Australia, with over 8 million residents [19]. State health policy is developed by the NSW Department of Health, while state government health services are administered by smaller units, the local health districts (LHDs) and speciality health networks (SHNs) [19]. Approximately one third of young Australians (12 to 25 years) make up 16.5% of the overall NSW population [16]. Prominent health issues for young people in NSW include mental health, suicide, chronic conditions, disability, obesity, accident and injury, sexual and reproductive health, and risk behaviours including substance misuse [16]. Socioecological factors intersect to play a complex role in determining health outcomes for young people in NSW [16, 17]. Both structural and cost factors impact access to care, particularly for marginalized youth [16]. Young people at higher risk of poorer health outcomes in NSW include (but are not limited to) Aboriginal and Torres Strait Islander peoples, refugees or newly arrived migrants, those who are experiencing or at risk of homelessness, those who are sexuality- and/or gender-diverse (lesbian, gay, bi, trans, queer, intersex [LGBTQI]), and those living in rural and remote areas [17, 18].

## The *Access* projects

Youth health has been an area of specific policy development in NSW for over two decades [18–21]. Youth health policies were released in 1998 [21], 2011 [18] and 2017 [20]. A strategic health plan for children, young people and families was also released in 2014 [19]. In 2000 and 2002, NSW Health commissioned two consecutive projects that informed development of the 2011 youth health policy [18], known as the *Access 1* project [22] and the *Access 2* project [23]. The *Access 1* project [22] was a needs analysis designed to elucidate when young people access healthcare services and whether they receive appropriate care. The *Access 2* project [23] utilized service provider perspectives to identify service models, principles and practices appropriate for supporting the health of young people. Importantly, formal KT processes were not “built into” these earlier *Access* projects.

In early 2015, NSW Health invited competitive tendering for the *Access 3* project to inform policy beyond 2016. The objective was to “gather policy-relevant intelligence about the experience of the young person when accessing and then navigating health care services. The priority perspective was the young person as consumer (and family where possible) with a key focus on marginalised youth” (Kang 2021, personal communication, 15 May) [24].

The *Access 3* project comprised four activities [16, 24–27]. These were (1) a large cross-sectional survey of young people residing in NSW (*n* = 01,416) [16], (2) a longitudinal qualitative study of a subsample of marginalized young people and their journeys through the health system over 6 to 12 months (*n* = 41) [26], (3) interviews with health professionals (*n* = 22) [27] and (4) a KT activity to support policy development [25].

The current paper reports on the KT activity (activity 4) of the *Access 3* project. The aim of this activity was to translate synthesized data from the first three *Access 3* activities into policy-ready recommendations to support development of a new state policy on youth health and well-being [20]. A key objective of this work was to bring together a broad and diverse group of stakeholders (including young people, policy-makers, health professionals and academics) in order to access knowledge, support interpretation of research results and draft policy recommendations [28]. Ensuring a youth voice within policy development was a key objective of the KT process. Here, we describe the KT approach for the *Access 3* project including participant satisfaction with this process and examine the current NSW Youth Health Framework [20] for evidence of research translation.

## Methods

### Design and theoretical approach

The KT activity consisted of a 1-day facilitated forum with invited stakeholders that was held in Sydney, NSW, in November 2016. The KT frameworks of Lavis et al. [6] and Grimshaw et al. [7] guided the forum design and the development of a data collection instrument to collect policy themes and recommendations. Lavis et al. [6] provide a framework that requires those responsible for KT (in this case the *Access 3* team, stakeholders and policy-makers) to consider the following five questions:

- What should be transferred?
- To whom should research knowledge be transferred?
- By whom should research knowledge be transferred?
- How should research knowledge be transferred?
- With what effect should research knowledge be transferred?

Grimshaw et al. [7] expanded on this framework to ensure that barriers to and facilitators of successful KT are considered when answering each of the above questions.

### Procedures

The planning, management and execution of the KT activity involved a number of key steps including (1) synthesis of *Access 3* research evidence, (2) pre-forum planning and development of data collection tools, (3) recruitment of forum participants, (4) forum presentations, (5) small-group discussions, (6) forum evaluation, (7) synthesis and submission of policy recommendations, and (8) post hoc examination of policy translation. Throughout the KT process, the members of the *Access 3* team took on the role of knowledge brokers [7, 8], facilitating communication and building relationships between stakeholders (i.e. young people, clinicians and researchers) and the end users of evidence (i.e. policy-makers).

### Synthesis of *Access 3* research evidence

Research evidence to be translated came from the findings of the first three *Access 3* project activities (i.e. quantitative results from the cross-sectional survey and qualitative data captured from marginalized young people and health professionals) [16, 17, 26, 27]. The considerable breadth and amount of research data generated from these activities necessitated a synthesis of key findings to provide direction for KT efforts. To achieve this, the chief investigators conducted preliminary analyses of the data from *Access 3* activities 1, 2 and 3.

Quantitative analysis of survey data (activity 1) was conducted using SPSS version 24 software [30], and qualitative data from activities 2 and 3 were subjected to preliminary thematic analyses. Synthesis of findings from all three activities took place iteratively among the chief investigator team. Quantitative analysis provided new information about barriers to access, the use of digital technology in help-seeking and attitudes towards navigating the health system. These quantitative data were presented alongside themes and illustrative quotes from young people and health professionals which gave deeper insights into how and why young people experienced health-seeking, accessing health services and moving around the health system. Eight key themes were derived from the data synthesis process. The final results of these analyses were published separately [16, 17, 26, 27] after the release of the NSW Youth Health Framework 2017–2024 [20] (Table 1).

**Table 1:** Synthesized research themes identified from *Access 3* activities 1, 2 and 3

Theme 1: Young people’s health literacy embraces our connected, digitally disrupted world
Theme 2: Traditional barriers remain but technology brings new opportunities for young people to connect and engage with services
Theme 3: Health system navigation must be assertively supported
Theme 4: Engagement in healthcare is about people and positive interactions
Theme 5: Young people perceive and experience multiple prejudices
Theme 6: Healthcare costs are high and ripple out
Theme 7: The ideal general practitioner has many desirable qualities but is hard to find
Theme 8: Reducing system demands and complexity would create a more efficient and straightforward experience for young people

### Pre-forum planning

*Access 3* chief investigators planned the KT activity in collaboration with two NSW Health senior policy professionals responsible for establishing the new NSW Youth Health Framework, youth consultants, an academic with expertise in KT and an experienced workshop facilitator. Building and maintaining strong relationships between these groups was prioritized over the course of the entire *Access 3* project. A forum agenda (see Text Box 1) was created for the day which featured allocated times for stakeholder presentations and small-group discussion workshops. A series of face-to-face meetings, as well as email and phone discussions, helped to refine the forum agenda.

**Table:** 

Text box 1: Workshop agenda	
Agenda	
9:30–9:35	*Welcome and acknowledgement of country*
	Presented by: Aboriginal traditional owner
9:35–9:45	*Why today is really important for young people*
	Presented by: Youth consultant
9:45–10:15	*About the Access 3 project*
	Presented by: Chief investigator (academic)
10:15–10:30	*Knowledge translation… thinking bigger and broader*
	Presented by: Policy expert (academic)
10:30–10:45	*Language explained…*
	Presented by: Young people
10.45–11:00	*NSW Youth Health Policy*
	Presented by: Policy-makers
11:30–12:30	*Presentation of research findings and responses from young people*
	Presented by: *Access* lead project officer and youth consultants
12:30–1:00	*Workshop groups: Question 1*
	1. Young people’s health literacy embraces our connected, digitally disrupted world
	2. Traditional barriers remain but technology brings new opportunities
	3. Health system navigation must be assertively supported
	4. Engagement in healthcare is about people and positive interactions
	5. Young people perceive and experience multiple prejudices
	6. Healthcare costs are high and ripple out
	7. The ideal general practitioner has many desirable qualities but is hard to find
	8. Reducing system demands and complexity would create a more efficient and straightforward experience for young people
1:30–3:30	*Workshops: Questions 2–5*
	1. Young people’s health literacy embraces our connected, digitally disrupted world
	2. Traditional barriers remain but technology brings new opportunities
	3. Health system navigation must be assertively supported
	4. Engagement in healthcare is about people and positive interactions
	5. Young people perceive and experience multiple prejudices
	6. Healthcare costs are high and ripple out
	7. The ideal general practitioner has many desirable qualities but is hard to find
	8. Reducing system demands and complexity would create a more efficient and straightforward experience for young people
3:40–4:00	*Synthesis of the day and wrap-up*

Another aspect of pre-forum planning was the development of data collection tools for use on the day of the KT forum. First, questions from the KT frameworks of Lavis et al. [1] and Grimshaw et al. [2] were adapted to suit the context of the KT workshop (see Table 2). These were then used to develop a data collection template to guide small-group discussion workshops and collect data on ideas for KT of evidence into policy recommendations discussed during the KT activity (see Additional file 1).

**Table 2:** Workshop questions template adapted from Lavis et al. and Grimshaw et al.

Questions posed at workshop	Questions from KT frameworks
How does the group understand and support this theme?	What should be transferred? [1]
Which groups or locations or healthcare settings is this theme particularly relevant for?	To whom should research knowledge be transferred? [1]
Who would need to be involved in its implementation?	By whom should research knowledge be transferred? [1]
How can this theme be implemented?	How should research knowledge be transferred? [1]
What difference will this make?	With what effect should research knowledge be transferred? [1]
What would support implementation?	What are the barriers and facilitators to successful knowledge translation? [2]

Next, a brief self-report survey was created to evaluate participant satisfaction with the KT forum. The seven-item survey measured the extent to which participants felt they were able to contribute meaningfully to the task of translating research, and the extent to which the facilitation, directions and activities of the forum aided development of policy-ready recommendations. A general remarks section was also provided for comments on forum organization, attendees, structure and processes, outcomes and overall perspectives.

### Recruitment of forum participants

A total of 64 stakeholders were recruited via direct email to participate in the forum. Stakeholders included young people, policy analysts, expert clinicians, researchers, community advocates, and senior staff from NSW Health (see Table 3). Forum members were purposively sampled based on their role (youth consultant, policy-maker, clinician, manager, academic, other), health system level (primary, secondary, tertiary), health service type (public, private, nongovernmental organization), and service focus (general population vs specific marginalized groups), and geographical location (metropolitan vs rural). Approximately a third of forum attendees had previously been involved with the *Access 3* project via reference groups (28), including chief investigators (7), associate investigators (5), urban reference groups (5), rural reference groups (5) and youth consultants (6). Stakeholders that accepted an invitation were sent a research report prior to the day of the KT forum which outlined the eight themes synthesized from earlier *Access 3* research activities.

**Table 3:** Workshop attendees by group

Group	*n*
NSW health services	22
Academia	15
Policy	14
Young people	8
General practitioners	4
Mental health (Headspace)	1
Total	64

### Forum presentations

The KT forum was held in Sydney, NSW, in November 2016. The morning session of the KT forum involved several presentations focused on translating findings from the first three *Access 3* activities (see Text Box 1). Presentations included an acknowledgement of country and traditional owners of the land, a presentation from a youth consultant on their perspective and experiences of the health system, and a presentation from the chief investigator on the *Access 3* project including background, examples of prior policy translation, and an overview of *Access 3* project design and results. Next, a NSW government policy-maker delivered a presentation on current health policy and the context for the new policy under development. A health policy academic then gave an overview of KT processes and explained the key tasks for forum participants (i.e. to consider *Access 3* findings and work collaboratively to build policy solutions). Next, a research officer (also a chief investigator) presented preliminary research findings around the eight key themes for small-group discussion. Finally, young people shared their reflections on the presentation of themes. Key principles for the forum programme included credibility of the research to the stakeholders, end users being active contributors to translation of findings, and structures being there to support the mobilization of knowledge.

### Small-group discussions

The afternoon session of the KT forum featured small-group discussions that were utilized to develop policy recommendations (see Text Box 1). Forum participants were pre-allocated to one of eight tables (groups), where they would discuss one of the eight key research themes synthesized from the *Access 3* project (see Table 1). The data collection template (see Additional file 1) was used to guide discussions and provided a range of questions and prompts based on the KT frameworks of Lavis et al. [1] and Grimshaw et al. [2]. Each group featured a range of stakeholders (*n* = 8) including at least one young person and a group facilitator who was an *Access 3* chief or associate investigator.

Initially, each group was asked to discuss their understanding and support for their allocated theme (see Additional file 1). Workshop members were encouraged to share their perspectives and discuss the importance of issues covered by the overarching theme. Next, each group was asked to consider the health settings that were relevant to their allocated theme and potential factors that could impact implementation. Participants were encouraged to consider possible conflicting priorities and current policy commitments, and to consider how their recommendations might be used and the potential audiences. Participants were not restricted to discussion of state-level actions and were able to consider recommendations at the federal policy level if they wished. Participants were encouraged to discuss conflicting opinions within group workshops, with facilitators ensuring that debate was conducted in an inclusive and respectful manner.

The workshop groups were instructed to utilize the data collection template to develop and refine three top policy recommendations related to their theme and to share these with the entire forum audience. These ideas were captured on large sheets of paper and displayed on the walls of the conference room. As a final activity, each KT forum participant was given five red dots to stick on the sheets of paper as a vote for their top ideas for implementation. Participants were not restricted to voting on the theme/workshop group they participated in, but rather, could vote on any of the ideas displayed on the walls.

### Synthesis and submission of policy recommendations

Synthesis of the policy recommendations developed during the KT forum was conducted by the chief investigators of the *Access 3* team in the weeks following the KT forum. The investigators performed content and thematic analysis of the responses captured on the data collection templates and the sheets of paper that captured the votes on policy recommendation ideas from each forum group. The policy recommendations with the highest number of votes in each of the themes became the final list for the *Access 3* investigators to work with. The recommendations established through the KT activity were presented to NSW Health in the form of a presentation and supporting report. The policy-makers responsible for authorship of the NSW Youth Health Framework 2017–2024 received copies of both the report and the presentation [20].

### Post hoc examination of policy translation

A post hoc examination of the NSW Youth Health Framework [20] was conducted in November 2020 to identify potential translation of *Access 3* forum recommendations into health policy actions for NSW. This exploratory exercise consisted of the lead author examining content from the published health policy and mapping this to the recommendations created through the KT workshop. This was achieved through thematic coding of the NSW Youth Health Framework [20] in NVivo 11 [29] using the themes developed from the 2016 KT workshop.

## Results

### Workshop themes and policy recommendations

Six broad themes were generated from the synthesis of overlapping ideas between forum workshop groups. These themes were (1) technology solutions, (2) integrated care and investment to improve capacity, (3) Medicare structures, (4) workforce capacity-building, (5) youth participation and (6) quality systems. A total of 25 individual policy recommendations were established across these six themes. Table 4 outlines these recommendations and the number of votes each individual recommendation received. The most popular policy recommendations related to the hiring and development of youth health workers, involving young people at the heart of decision-making, and the importance of youth health indicators and screening. Notably, some of these recommendations related to Australia’s universal health insurance (Medicare), which is federally administered and thus outside the scope of NSW state policy.

**Table 4:** Workshop policy recommendations

Theme	Top implementation ideas	Votes
Theme 1: Technology solutions	Streamlined portal: promotion through social media marketing, helping young people navigate efficiently and effectively, combining all websites and general health information	10
	Apps to locate general practitioners and allied health professionals via postcode that filter by cost, hours, rating, bulk billing, LGBTQI-friendly, map and travel info	9
	Optimize traffic to government websites through marketing e.g. paid media on Facebook and Google search	8
	Health online pathways (primary care networks): flowchart/platform-specialized advice for this group, local/referral pathways, promotion with youth workers and practices, consumer flowchart for the young person	8
	Broadening access to general practitioners: via technology e.g. YouTube education videos, common consultation, app chat	5
	Online directory of services for young people including key information (e.g. bulk billing) and youth ratings	5
	Infrastructures: access, quality, cost with cross-sector partnerships e.g. telcos	4
	Cultural change through (1) empowerment of young people through access to information and education, (2) youth-friendly services: campaigns (stickers), websites (cost, hours of transport, bulk billing, minimum standards, service mapping), and (3) government valuing youth health, funding, equity in access to services across state	4
Theme 2: Integrated care and investment to improve capacity	Establish youth medical assessment team (in local health districts) that parallels geriatric services: nurse practitioner tasked with navigation, salaried medical officer	14
	Shared care model: Headspace-accredited youth-friendly general practitioners, percolative health systems	10
	Emergency department: 24/7 targeted structures that link back to youth medical assessment team	5
	Integrated care: primary healthcare, general practice and hospital sectors “primary healthcare team”, pool funding, commit to the time to do this	4
	Capacity: service- and systems-level investment to deliver better and integrated services	3
	Cross-sectoral work: training, planning, internal and external to health	3
Theme 3: Medicare structures	15+ youth check: incentive for general practitioners and young people, digital pre-screen (red flags), long consultation item navigation universal access funnel, low need, high need, very high need	15
	Change in Medicare model: item number for youth health assessment, youth-accredited general practitioners	14
	Medicare item numbers for youth health: making the case for appropriately funding youth-integrated services, young people learning how to navigate health	9
Theme 4: Workforce capacity-building	Trained youth worker: advocacy, facilitator, navigating, training and education to practices and professionals	17
	Build capacity of youth workforce (health, Aboriginal medical service, justice, education) to embed health literacy in core business	7
	Ongoing professional development for all health providers: youth-friendly services training, especially for marginalized young people (multiple prejudices), current, up-to-date to our climate	4
	Training, education and resources with continuing professional development points for health professionals (including cultural and gender sensitivity) and key references like youth services and schools to promote engagement at first contact with health services	4
	Capabilities: knowledge and skills for young people, professionals, parents, educators and policy-makers	3
Theme 5: Youth participation	Young people at the heart of decision-making—“Nothing for us without us”	17
	User-centred approach to research, design, implementation and evaluation (youth participation and professionals)	4
Theme 6: Quality systems	Best-practice youth health indicators included in standard accreditation systems e.g. general practice/primary care accreditation, public health system accreditation	15

### Forum evaluation

Forty-five forum participants (70.3%) completed the forum evaluation survey. The majority of respondents thought the workshop activities meaningfully contributed to the task of translating research into NSW youth health policy possibilities (93.3%) and felt that they were able to contribute to the small-group discussions (88.8%). Participants who reported being only partly able to make contributions to small-group discussions (*n* = 4) were asked to provide suggestions for how to improve group processes. Responses included ensuring facilitators held tighter adherence to questions posed, more efficient moderating of dominant group members, greater diversity of health disciplines on the table and ability to provide greater contribution to some of the topic areas discussed at other tables.

General comments from attendees indicated that the forum was well structured and facilitated, and featured knowledgeable attendees and good engagement of young people affected by policy. The absence of Aboriginal youth representation was mentioned (however, one of the youth representatives did identify as Indigenous), as was the desire for greater time for strategy development and intergroup feedback. When considering outcomes of the day, comments were positive, with participants having hope for their work being translated into concrete policy actions. Table 5 summarizes the quantitative results of the survey, which indicates high overall satisfaction with the KT forum and small-group workshops.

**Table 5:** Workshop evaluation responses

	Strongly agree	Agree	Unsure	Disagree	Strongly disagree	No response
Overall, today’s process allowed me to contribute meaningfully to the task of translating research into NSW youth health policy possibilities	18	24	0	0	1	2
Overall, the facilitation, directions and activities of the day helped us achieve our goals	19	22	0	0	1	3
This part of the agenda [group feedback and wrap-up] was useful for understanding what other groups had talked about and being able to make final contributions	12	24	7	0	0	2

	Yes	No	Partly	No response
Did you feel like you were able to make the contribution you wanted to in the small group?	40	0	4	1

What were the most useful parts of the agenda leading up to the small groups? (top responses)	*n*					
a. Research findings and young people responses	15					
b. Policy translation	9					
c. About the project	5					
d. All presentations	5					
e. Why today is really important for young people	4					

What part/s of that process were least useful from your point of view? (top responses)	*n*					
a. Research findings and young people responses	5					
b. Policy translation	3					
c. Hearing things already sent in writing	3					

### Post hoc examination of policy translation

The NSW Youth Health Framework 2017–2024 [20] was launched by the NSW Minister for Health on 6 July 2017 at the Australian Association for Adolescent Health (AAAH) Youth Health Conference [31]. This served as an opportunity to inform and raise awareness of the policy with relevant stakeholders, including some of those who took part in *Access 3* activities.

Post hoc exploratory document analysis of the NSW Youth Health Framework [20] provided evidence of translation of *Access 3* recommendations into policy statements from the NSW Youth Health Framework [20]. Indeed, page 3 of the framework [20] states that the policy “takes account of relevant research and evidence including the *Access* research studies 1, 2, 3 which explore young people’s experiences of accessing and navigating health services in NSW”.

Table 6 provides examples of specific policy recommendations from the KT forum next to relevant policy statements from the framework. Importantly, general themes from the research (such as supporting young people’s health system navigation) were also evident in the policy.

**Table 6:** Research-to-policy translation examples

Theme	Access 3 policy recommendation	NSW Youth Health Framework
Technology solutions	“Streamlined portal: promotion through social media marketing, helping young people navigate efficiently and effectively, combining all websites and general health information”	“Maximise opportunities to provide up-to-date and accessible online information for young people about health services including who they are for, how to access them, what they do, and costs involved.” “Support health services to adopt appropriate technology, including telehealth, Apps, mobile technology and social media, to support access to services and engage and seek feedback from young people.”
Technology solutions	“Apps to locate general practitioners and allied health professionals via postcode that filter by cost, hours, rating, bulk billing, LGBTQI-friendly, map and travel info”	“The Framework sets an expectation that NSW Health services for young people will use available electronic and mobile communication methods, and that online information is appropriate and meaningful. Further opportunities will be explored to develop and implement appropriate technology as part of service delivery, particularly to support young people living in rural and remote areas.”
Technology solutions	“Broadening access to general practitioners: via technology e.g. YouTube education videos, common consultation, app chat”	“Further opportunities will be explored to develop and implement appropriate technology as part of service delivery, particularly to support young people living in rural and remote areas.” “Support health services to adopt appropriate technology, including telehealth, Apps, mobile technology and social media, to support access to services and engage and seek feedback from young people.”
Workforce capacity-building	“Build capacity of youth workforce (health, Aboriginal medical service, justice, education) to embed health literacy in core business”	“Work with partner agencies to support and provide health promotion information, programs and services, and create healthy environments for young people in line with state and local priorities that support healthy living, physical and mental wellbeing, health literacy, harm and demand reduction, sexual and reproductive health, and injury prevention.”
Workforce capacity-building	“Capabilities: knowledge and skills for young people, professionals, parents, educators and policy-makers”	“Workforce capacity to provide responsive care to young people that promotes safety, welfare and well-being”
Youth participation	“Young people at the heart of decision-making—‘Nothing for us without us’”	“Young people’s health needs are responded to; they receive quality healthcare and are supported to make informed decisions.”
Integrated care and investment to improve capacity	“Capacity: service and systems level investment to deliver better and integrated services”	“NSW Health will strengthen relationships with other health services and cross sector partners to provide integrated and coordinated care.”

## Discussion

The current paper outlines the KT activities of the *Access 3* project, which were specifically designed to inform NSW youth health policy. We believe the design of this translation activity represents a step forward in Australian youth health policy-making, as it brought together a range of different perspectives including those of young people, academics, health workers and policy-makers to develop policy recommendations using a strong evidence base on youth health issues and a theoretically derived KT framework. Formal KT processes have not been “built into” the development of prior youth health policies in NSW, and we feel the methods described here provide a strong platform for future efforts to support evidence-informed policy-making in this arena.

Specifically, the KT forum led to the development of six policy themes of areas for policy action with 25 specific policy recommendations proffered. Participant satisfaction with the KT forum was high and, importantly, the policy recommendations from the workshops can be evidenced within the subsequent NSW Youth Health Framework [20]. These results speak strongly to the success of building considered approaches to policy development and KT.

There are several aspects of the KT forum that likely contributed to this success. Central to these is the utilization of the KT frameworks of Lavis et al. and Grimshaw et al. [6, 7]. These frameworks provided structure to the planning, execution and evaluation of the KT forum including the specific workshop activities and the knowledge dissemination strategies utilized. We therefore focus subsequent discussion around the key questions posed within these frameworks.

### What should be transferred?

Effective KT requires quality evidence [6, 7]. Whilst researchers and research organizations, field experts, clinicians, consumers, peak bodies and government bodies are often good sources of information, the evidence they provide is not always fit for direct policy translation [5, 6, 32]. The best evidence to support policy changes comes from pooled research knowledge in the form of systematic reviews or from research studies that are sufficiently large and targeted at specific policy questions [5–8]. The relevance and timeliness of evidence are particularly important influences on knowledge uptake [8, 33]. Presenting evidence in the form of “ideas” rather than research data also improves the likelihood of translation, particularly when working with diverse groups and nonacademic audiences [6].

In relation to the *Access 3* project, the evidence established through activities 1 to 3 is of high quality and relevance, as the activities were designed specifically for answering policy questions relevant to youth health [24, 25]. The demand-driven nature of the tendering process for the *Access 3* work meant that this knowledge was sought after by the policy-makers and developed in a timely manner with policy-makers involved in the planning, execution and translation aspects of the project. Also, the translation forum allowed the research team to present the findings from activities 1 to 3 in the form of “research themes” or “ideas” and to transform these into actionable policy recommendations that were broadly aligned with the remit of NSW Health.

### To whom should research knowledge be transferred?

The target audience for KT activities must be clearly identified to ensure success [6, 7]. Having a well-defined target group allows knowledge translators to better understand the types of decisions and decision-making environments that exist for the particular target, which in turn allows for the tailoring of KT strategies [6]. For the current activity, the target audience was defined as policy-makers from the NSW Ministry of Health. The goal of the KT workshop was for these policy-makers to be aware of and utilize the findings and policy recommendations from the *Access 3* KT forum to inform policy development for the NSW Youth Health Framework [20]. Consideration of the political and organizational constraints that face NSW Health policy-makers was built into the planning, execution and evaluation of the KT activities.

A key aspect of this approach was gaining an understanding of the NSW policy-making environment and the factors that influenced decision-making processes within it. Working with policy-makers throughout the research and KT process helped build this collaborative partnership. Importantly, the *Access 3* research and KT forum sat in the context of the broader relationship with the policy-makers, where researchers sat on a policy development reference group and gave comments on the policy and separately presented the research findings to policy committees.

### By whom should research knowledge be transferred?

Effective KT requires a credible messenger to deliver evidence to target audiences [6–8]. Individuals (e.g. health professionals, researchers or consumers), groups, organizations and the healthcare system can all act as messengers for KT activities focused at policy-makers [7]. Whilst building credibility with this target audience may be difficult and/or time-consuming, it is an important aspect for effective KT [6, 7].

Throughout the KT process, the members of the *Access 3* team took on the role of knowledge brokers [7, 8, 32, 34, 35] working as intermediaries to build important connections between evidence suppliers (i.e. researchers, clinicians and young people) and evidence users (i.e. policy-makers). This process featured iterative and bidirectional communication between stakeholders and policy-makers to promote trust and greater understanding [35].

The KT activity utilized a broad stakeholder collaborative to deliver our message to the NSW Ministry of Health. We utilized the voices of expert clinicians and impartial researchers, as they are shown to be authoritative messengers for the development of evidence-informed health policy [6, 8]. We also included policy-makers in the KT forum and research processes to ensure that the collaborative had a sound understanding of the policy process and the context surrounding NSW Health policy agendas. We also made sure to actively include young people in the policy development process (as well as throughout the entire *Access 3* project).

To date, efforts to include young people in the development of policy remains variable across settings and portfolios, with inclusion influenced by a range of political and ideological factors [36]. Furthermore, when young people have been involved in the development of policy, this has often been limited to participating in rigidly structured consultations that have featured top-down approaches to policy development [36, 37]. Such efforts have been labelled “tokenistic” in their approach [36].

To counter this, we prioritized the active inclusion of young people in the formulation of specific policy recommendations for the youth health policy. The *Access 3* project team shared a commitment to sustained and continuous youth engagement and encouraged KT stakeholders and their organizations (including the NSW Ministry of Health) to also value this engagement. Embedding such values throughout the KT process was considered an important design principle for building effective stakeholder engagement [28].

### How should research knowledge be transferred?

A key explanation for the research–policy gap is the disparate and asynchronous responsibilities, priorities and processes that exist within the domains of research and policy [6–8, 10, 15, 35]. Research is typically investigator-driven and usually proceeds in a steady, methodical and linear fashion, with publication of research findings often prioritized over translation efforts [15]. In contrast, policy is often developed in a fast-paced, unpredictable environment that involves a raft of competing demands, priorities and stakeholders [6–8, 15]. Whilst policy is applied by nature, policy decisions may be influenced more by opinion and political ideals rather than unbiased empirical evidence. Developing evidence-informed health policy thus requires strong and deep collaborations between researchers and policy-makers [7, 15, 35]. Researchers are required to develop relevant, timely and helpful evidence that can be effectively translated into policy. Policy-makers must appraise available evidence, navigate entrenched political and economic interests, and balance these alongside the social acceptability of the policy they are tasked to deliver [38].

There is a growing evidence base to guide choice of KT strategies aimed at policy-makers [32]. Specific factors that facilitate research uptake include interactive engagement between researchers and policy-makers, and improved relationships and skills [8, 32]. KT is thus most effective when it starts early, builds support through champions and brokers, understands contextual factors, and is timely, relevant and accessible [32]. For the current activity, we utilized workshops involving a variety of stakeholders and built deep relationships over a period of time to provide formulated recommendations to government through an established pathway. The partnerships built between investigators, forum participants and NSW Health underpinned the strength of this translation approach.

### With what effect should knowledge be transferred?

When considering KT, it is important to determine how it is hoped that research knowledge will be used [6]. In a health setting, this may be getting a clinician to change their behaviour in the face of research evidence whereas, in a policy setting, the goal may be less concrete and may simply be to inform debate, especially given competing organizational and political factors [6, 7]. For the current activity, the overarching goal was to develop implementable policy recommendations that could be provided to the NSW Ministry of Health for consideration for inclusion in the youth health framework [20]. The fact that the research themes and recommendations provided to the ministry could be mapped onto policy items within the framework suggests that this approach was effective.

### Strengths and limitations

The KT activity presented here featured both strengths and limitations. A key strength is that NSW Health commissioned the *Access 3* project and KT forum, which likely had an impact on policy-maker buy-in. Demand-driven research is known to be more effectively translated [6, 7, 15, 35], and it is probable that engaging policy-makers would be more difficult when this is not the case. We believe that the KT frameworks and approaches outlined in this paper assisted the development of strong relationships and provide a strong model for collaboration between researchers and government that aligns with the WHO strategy on health policy and systems research [39].

A limitation of our approach is that it is difficult to obtain an objective metric of KT success. Whilst document analysis allowed the authors to map policy recommendations onto the NSW Youth Health Framework [20], this approach may be considered subjective and hence may over- or underestimate the impact of KT efforts. Whilst we acknowledge this limitation, the positive evaluation we received from policy-makers engaged in the workshop suggests that our approaches were indeed impactful.

Second, whilst the forum led to implementable policy recommendations, there were some recommendations that fell outside of the scope of NSW Health policy. Specifically, these recommendations were related to federally administered Medicare structures that can shape the role and function of general practitioners. Importantly, this issue was highlighted and discussed at the KT forum. It was underlined that there was an audience for these kinds of recommendations beyond the NSW Youth Health Framework. We believe that KT never ends in a closed system and that changes in one part of the overall health system will inevitably have flow-on effects throughout the health system. Future work could look at how the development of the NSW Youth Health Framework influenced and impacted the later development of policies across Australia at both a state and federal level.

Third, the required setup and timing of the forum meant some concessions had to be made. For example, the timing of the forum was due to policy-makers’ needs and not the researchers, and thus required the presentation of preliminary rather than final research results. Nevertheless, the final findings of the research matched the themes presented at the KT forum, which suggests that the impacts of timing were minimal in this case. Overall, we believe that the approaches used were appropriate and led to strong levels of engagement from stakeholders and robust recommendations for policy.

Finally, the current activity stopped short of analysing the underlying contexts, mechanisms or practices that led to policy translation or examining the actual implementation of policy recommendations that made their way into the NSW Health framework. This was considered beyond the scope of the *Access 3* project and KT process. Measuring the pathways and success of knowledge transfer beyond decision-making in the health policy realm is difficult, as the routes from which research-informed decisions translate into actual social, economic or health outcomes are complex [6]. Nevertheless, we recommend and would welcome future investigation focused on the implementation of youth health policies.

In summary, we believe that the utilization of KT theories and youth inclusion led to the successful transfer of evidence-based knowledge from the *Access 3* project into NSW Health policy. We would therefore encourage researchers from abroad to consider such approaches for the development of youth health policy within their respective states and countries. By actively engaging young people and utilizing theoretically supported KT frameworks, we can build more inclusive and appropriate health policies that promote the health of our younger generations. Within NSW, there is now a clear opportunity to examine the implementation of policy recommendations [40]. By conducting this research, we may better understand the contexts, mechanisms and outcomes surrounding policy implementation in the youth health space, which will provide a clearer picture of how evidence is translated into subsequent action.

## Conclusions

Bridging the research–policy gap is critical to ensuring that policy decisions are fair, equitable and based on a sound understanding of relevant issues. The current case study demonstrates an effective approach to the translation of research knowledge into policy recommendations utilizing established KT frameworks. Further research into the implementation of policy actions developed from these KT approaches is warranted.

## Supplementary Information

**Additional file 1.** Data collection template

## Data Availability

The datasets analysed during the current project are available from the corresponding author on reasonable request.
