# Supplementary material for: Building evidence into youth health policy: a case study of the Access 3 knowledge translation forum
Source: Health Res Policy Syst. 2022 Apr 20;20:44. doi: 10.1186/s12961-022-00845-y (PMC9022323; doi:10.1186/s12961-022-00845-y)
Supplement: Supplementary file 1 — Additional file 1. Data collection template [file 12961_2022_845_MOESM1_ESM.docx]

Supplementary file 1: Data collection template

**WORKSHOP THEME:** _

|  | Can you share your different perspectives on how you understand this theme?  How much support in the group is there for this theme to be incorporated into policy? | | |
| --- | --- | --- | --- |
| 1. How does the group understand and support this theme? |  | | |
|  | Please describe | Can you give us 2 - 3 examples? | What difference will this make? |
| 2. Which groups or locations or health care settings is this theme particularly relevant for? |  |  |  |
| 3. How can this theme be implemented?  – consider the barriers and facilitators |  |  |  |
| 4. Who would need to be involved in its implementation? |  |  |  |
| 5. What would support implementation?   - practical suggestions - consider innovative, creative strategies |  |  |  |
